# Measuring Surface Electromyography with Textile Electrodes in a Smart Leg Sleeve †

**DOI:** 10.3390/s24092763

**Published:** 2024-04-26

**Authors:** Federica Amitrano, Armando Coccia, Gaetano Pagano, Arcangelo Biancardi, Giuseppe Tombolini, Vito Marsico, Giovanni D’Addio

**Affiliations:** 1 Bioengineering Unit, Telese Terme Institute, Istituti Clinici Scientifici Maugeri IRCCS, 82037 Telese Terme, Italy; federica.amitrano@icsmaugeri.it (F.A.); gianni.daddio@icsmaugeri.it (G.D.); 2 Bioengineering Unit, Bari Institute, Istituti Clinici Scientifici Maugeri IRCCS, 70124 Bari, Italy; gaetano.pagano@icsmaugeri.it; 3 Tombolini Officine Ortopediche, 74121 Taranto, Italy; 4 Orthopaedics Unit, Bari Institute, Istituti Clinici Scientifici Maugeri IRCCS, 70124 Bari, Italy; vito.marsico@icsmaugeri.it

**Keywords:** e-textile, textile-based electrode, surface electromyography, EMG, wearable sensors, comfort rating scale, comfort assessment

## Abstract

This paper presents the design, development, and validation of a novel e-textile leg sleeve for non-invasive Surface Electromyography (sEMG) monitoring. This wearable device incorporates e-textile sensors for sEMG signal acquisition from the lower limb muscles, specifically the anterior tibialis and lateral gastrocnemius. Validation was conducted by performing a comparative study with eleven healthy volunteers to evaluate the performance of the e-textile sleeve in acquiring sEMG signals compared to traditional Ag/AgCl electrodes. The results demonstrated strong agreement between the e-textile and conventional methods in measuring descriptive metrics of the signals, including area, power, mean, and root mean square. The paired data *t*-test did not reveal any statistically significant differences, and the Bland–Altman analysis indicated negligible bias between the measures recorded using the two methods. In addition, this study evaluated the wearability and comfort of the e-textile sleeve using the Comfort Rating Scale (CRS). Overall, the scores confirmed that the proposed device is highly wearable and comfortable, highlighting its suitability for everyday use in patient care.

## 1. Introduction

Technological advancements have transformed the healthcare sector, with wearable technologies emerging as a key component of patient monitoring and health management [1,2]. These wearable devices, characterised by their constant connectivity, comfort, and discreet integration into daily life, are rapidly becoming indispensable tools in medical diagnostics and therapy [3,4]. The evolution from simple step counting to advanced monitoring of vital signs such as blood pressure and potential arrhythmias underscores the growing ability of wearable technologies to seamlessly integrate into our daily lives while improving access to healthcare and disease prevention [5]. The integration of wearable technologies extends from sports medicine, as demonstrated by Skazalski et al. [1], who used wearable devices to monitor the jumping load in elite volleyball players, to extreme conditions, where Chen et al. [2] developed methods to detect heat stroke. Similarly, Dooley et al. [6] compared and validated key consumer devices for measuring exercise intensity, highlighting the role of wearable devices in fitness and health tracking.

The use of wearable devices in healthcare, such as smartwatches, electronic bracelets, and sensor-embedded garments, represents a move towards patient-centred care. These devices enable real-time monitoring of crucial health metrics such as heart rate, blood pressure, and body temperature, facilitating immediate medical intervention when necessary [3,7].

Outstanding research has highlighted the potential of wearable technologies in the medical field, particularly their application in continuous health monitoring and disease prevention [8]. Wearable devices offer a unique combination of convenience, real-time data analysis, and personalisation of medical treatments, thereby revolutionising patient care and disease management.

However, a major challenge in the field of wearable technology is the integration of sensors with everyday clothing to improve usability and comfort. This challenge has led to the development of Electronic Textiles (e-textiles) or Smart Textiles which seamlessly integrate electronic components into textile materials and use innovative materials such as graphene to demonstrate the effectiveness of e-textiles in continuous health monitoring [9,10,11]. Despite their potential, challenges related to signal distortion and reduced stability with repeated washing remain critical issues to be addressed, highlighting the need for further advances in the field [12]. E-textiles represent a groundbreaking advancement, as they react and adapt to environmental stimuli through the integration of smart materials into their structure. This innovation presents several advantages over traditional electronic devices, including direct contact with the skin, flexibility and adaptability to the human body’s contours, and cost-effectiveness thanks to reusability and washability that is comparable regular clothing [8].

Recent studies, such as the systematic review of e-textiles in biomedical applications by Cesarelli et al., have highlighted the role of wearable biosensors and fabric-based devices in health monitoring and disease management, offering new paradigms in patient care [13]. Their comprehensive review illustrates the various applications and potentialities of e-textiles in various medical settings, reinforcing the importance of these technologies in improving patient comfort and autonomy in health management. However, the same review identified a common limitation regarding the small study populations used to test these novel devices. Typically, devices are tested on a single volunteer. This is likely correlated with the other relevant issue, which concerns the early stage of development of the technologies presented in the literature thus far.

sEMG is of great importance due to its wide range of applications and benefits. Among other interesting applications, during biomechanical analysis of movements and practical rehabilitation it can provide a simple and objective quantitative assessment of muscle function with high information content [14,15,16]. sEMG has emerged as a suitable application of e-textile technology considering the need to detect the muscle action potentials with electrodes directly on the skin [17]. However, the main challenge remains developing electrodes and devices that are accurate and reliable while also being comfortable and easy for patients to use. Recently, attention has shifted towards the use of flexible materials and conductive fabrics, which promise to overcome many of the limitations of traditional electrodes. Recent studies have explored the use of non-invasive flexible electrodes for sEMG acquisition, exploiting designs inspired by biological structures to improve adhesion and reduce interference during signal acquisition [18]. For example, the creation of adhesive microstructures on the surface of electrodes, inspired by natural mechanisms such as tree roots or marine organisms, has been shown to significantly improve both the adhesion and extension capability of electrodes. Another significant area of research concerns the use of conductive fabrics such as Conductive Composite Silicone Material (CCSM), which have demonstrated superior performance compared to screen-printed materials in terms of durability and resistance to deformation during use [19]. Conductive plating on flexible fabrics offers an interesting alternative, with a conductive coating on each individual fibre of the fabric to improve strength and flexibility.

In addition to these innovations in materials and fabrication techniques, it is crucial to consider the integration of these advances with wearable devices that can be easily adopted by users. This approach requires a focus on functionality and reliability as well as on ergonomics and user comfort, aspects that are crucial to ensure the wide adoption and sustained use of these devices.

In this context, several approaches to creating prototype e-textile devices have been identified in the literature. For instance, Ohiri et al. [19] proposed a modular suit designed to extensively measure muscle activity, with sensors on key muscle groups such as the torso, arms, legs, and back. Similarly, Goncu-Berk et al. [20] developed prototype t-shirts with varying sleeve lengths made of stretch polyester knit fabric and e-textile electrodes sewn with conductive thread. Alizadeh-Meghrazi et al. [21] proposed a sleeve covering the entire forearm that integrates knitted textile electrodes using conductive silicone rubber-based filaments. Ozturk and Yapici [22,23] analysed the performance of wearable graphene-based electrodes in monitoring the muscular activity the upper and lower limbs, showing acceptable Signal to Noise Ratio (SNR) values. Similar results regarding graphene-based electrodes were obtained by Awan et al. [24] in a cohort of eight healthy controls. Other studies have explored different structures and materials for textile electrodes, as shown by Katherine Le et al. [25], who analysed the effect of different types of conductive pastes and textile electrode structures on biopotential monitoring performance. Furthermore, Milad Alizadeh-Meghrazi et al. [26] examined the importance of skin–electrode impedance and embroidery technique in the effectiveness of sEMG textile electrodes. A different approach was followed by Choudry et al. [27], who used flexible conductive threads stitched on fabric to design textile-based piezoresistive sensors embedded inside a garment to measure muscle activity based on the small pressure changes exerted by muscles. These different approaches highlight the continuing innovation in the design and application of e-textiles, as further demonstrated by the previously mentioned systematic literature review [13] highlighting the expanding scope and capabilities of e-textiles in the biomedical field.

Furthermore, a topic of debate in scientific discussions concerns the size of the electrodes. Kim et al. [28] reported that electrodes with diameters of 20 and 30 mm outperformed those with smaller dimensions in terms of SNR and baseline noise. This finding is consistent with other studies that have shown lower electrode–skin impedance and better sEMG signal quality for electrodes with a larger surface area [29,30,31,32]. Despite these advantages, it is important to consider that increasing the size and thereby reducing the inter-electrode distance can lead to an increase in sEMG cross-talk and production complexity. The device presented in this work embeds small-diameter (10 mm) circular electrodes with increased skin contact pressure to overcome this issue. In fact, the studies by Kim et al. [28] and Taji et al. [33] suggest that increasing the clothing pressure on skin can lead to better performance with smaller electrodes. An increased contact area can be achieved by adjusting the tightness of the clothing using arm or leg sleeves [29] or by inserting pads or foams of various thicknesses between the electrodes and the substrate fabrics [34,35]. We considered both the solutions to improve the pressure on the electrodes; the proposed device is an adjustable sleeve made of elastic fabric, and the electrodes are located on foams fixed on the substrate fabric. On the other hand, excessive increases in the pressure of the device on the body district can cause discomfort and pain in the wearer, as reported by An et al. [29]. Therefore, in this study we assessed the wearability and comfort levels of the device by means of the Comfort Rating Scale.

The focus of this manuscript is on the development and validation of a novel wearable device, specifically, a textile leg sleeve with e-textile embedded sensors for sEMG. The proposed textile sleeve aims to provide a non-invasive, comfortable, and efficient tool to capture sEMG signals from specific muscles of the lower limb during gait in order to contribute to improved patient care and monitoring [17,36]. The sEMG sleeve is developed for integration into an ankle–foot orthotic device for patients with ankle dorsiflexion deficits. These supports have positive effects on walking [37]; however, it is of interest to assess the function of the muscles involved (i.e., the anterior tibialis and lateral gastrocnemius), which are the principal muscles responsible for foot dorsiflexion. Analysis of their activity, particularly during walking, can help to to improve biomechanical modeling of walking with the aim of diagnosing and monitoring clinical conditions [38]. This manuscript provides a comprehensive analysis, beginning with the design requirements and specifications of the e-textile band, followed by the fabrication process of the e-textile sleeve. Insight into the validation of the device using statistical methods on a study population of eleven healthy volunteers is presented, and the results of the study are reported and discussed with reference to the present scientific literature. This research aims to demonstrate the feasibility and effectiveness of wearable devices based on e-textiles in medical diagnostics and patient monitoring, which could pave the way for future innovations in healthcare technology.

## 2. Materials and Methods

### 2.1. Textile Sleeve for sEMG

The wearable device for acquiring sEMG signals is represented by an adjustable sleeve with removable e-textile electrodes, designed to create an adaptable device for various leg sizes that can be washed and reused. The electrodes for detecting sEMG signals are connected at specific points on the elastic sleeve, which is worn below the knee to capture signals from the anterior tibial and lateral gastrocnemius muscles.

The device is made of elastic fabric with a rectangular structure. The short ends are closed on themselves with an adjustable hook closure. This allows for sleeve adjustment and perfect adherence to various calf sizes. The initial structure is a rectangle measuring 300 mm in length and 240 mm in width, which is then folded and sewn onto itself to achieve a rectangle measuring 300 mm in length and 120 mm in width. Hook closures are then sewn onto the lateral edges of this rectangle. Regarding the outer side of the sleeve, six clips with a diameter of 13 mm are sewn for connection to the acquisition system. These clips are of the same size as those on standard Ag/AgCl electrodes used for biosignal collection, ensuring compatibility with sEMG acquisition systems.

For the inner side, which adheres to the calf, six clips with a diameter of 15 mm are sewn, onto which removable electrodes are applied. The electrode placement follows the Surface Electromyography for the Non-Invasive Assessment of Muscles (SENIAM) guidelines for sEMG detection of the anterior tibial and lateral gastrocnemius muscles [39]. The clips on the inner and outer sides of the sleeve are electrically connected to establish a connection between the electrode and the acquisition system.

**Figure 1:**
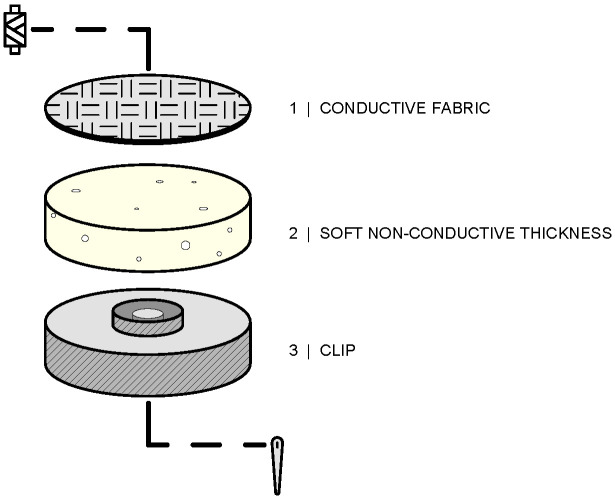
The textile electrode consists of three elements: a textile fabric placed on a non-conductive pad and then sewn to a metal clip.

Four removable electrodes with a diameter of 10 mm are used for acquiring the electromyography (EMG) signal from the two muscles of interest, along with two reference removable electrodes with a larger diameter (15 mm). The textile electrodes are made from Silver Fiber Knitted Fabric (Suzhou Yu Gao Radiation Protection Technology Co., Ltd., Suzhou, China), a conductive knitted fabric with a resistance of less than 1 Ω per foot in any direction across the fabric, and are wrapped in a soft non-conductive thickness to improve adhesion to the skin, then sewn onto a clip (Figure 1). This design allows for the electrodes to be removed when the acquisition is complete, allowing the sleeve to be washed.

### 2.2. Experimental Setup

The study involved eleven healthy volunteers (eight women and three men, age 25.7±1.7, height 168.3±5.9 cm, weight 62.2±5.9 kg) and evaluated the performance of the e-textile sleeve for sEMG in comparison to Ag/AgCl electrodes. The evaluation was performed by comparing the performance of the two types of electrodes in detecting sEMG signal characteristics during Maximal Voluntary Isometric Contraction (MVIC) of the lower limb muscles considered in the analysis. MVICs are commonly used, as they allow for comparison of muscle activity levels across muscles, tasks, and individuals while limiting the inhomogeneity due to intrinsic and extrinsic factors [40].

**Figure 2:**
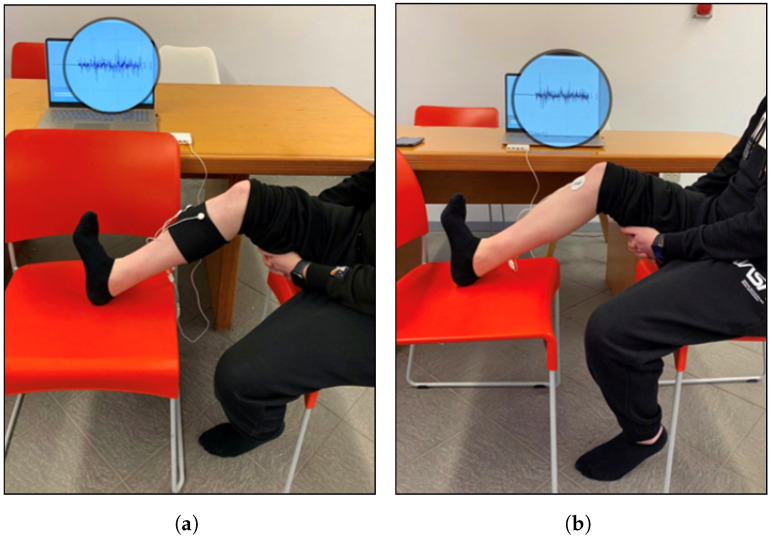
Experimental setup for acquisition of anterior tibialis EMG signal by (**a**) band with e-textile electrodes and (**b**) conventional pre-gelled electrodes.

**Figure 3:**
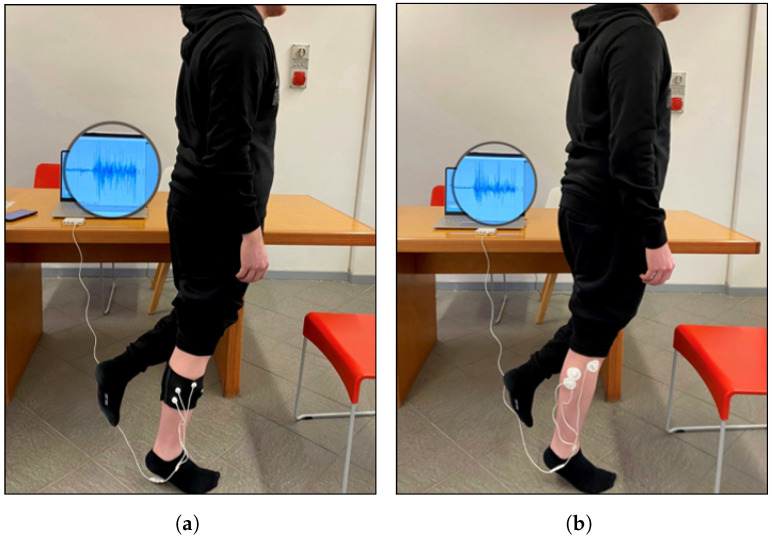
Experimental setup for acquisition of lateral gastrocnemius EMG signal by (**a**) band with e-textile electrodes and (**b**) conventional pre-gelled electrodes.

Each volunteer performed two measurement sessions, one with the textile sleeve and one with the standard electrodes, in random order to avoid ordering effects on the results. The session protocol involved the subject performing five MVICs, each lasting 5 s, with a rest of 30 s between contractions [40]. Specifically, EMG signals from the two target muscles, namely, the anterior tibialis and lateral gastrocnemius, were recorded separately. The acquisition was conducted with the assistance of a timer. When the timer was started, the subject remained in the resting position for 30 s. After the rest period, a start signal was given to the subject, who performed the task and the maximum isometric contraction for 5 s. Subsequently, the subject returned to the resting position and the protocol was repeated in the same manner for five cycles. For acquisition of the EMG signal from the anterior tibialis (Figure 2), the subject was seated and supported the leg, with the ankle joint in dorsiflexion and the foot in eversion without extension of the big toe (sensor locations: tibialis anterior; see the SENIAM guidelines). The MVIC of the lateral gastrocnemius (Figure 3) was obtained with the subject assuming an upright unipodal position, with the knee fully extended (0°) and the ankle in maximum plantar flexion [40]. Participants repeated the protocol with both limbs. Three out eleven participants had minor impairments of the left leg which affected the correct performance of the tasks. The total number of trials analysed was 19.

### 2.3. EMG Acquisition and Feature Extraction

**Figure 4:**
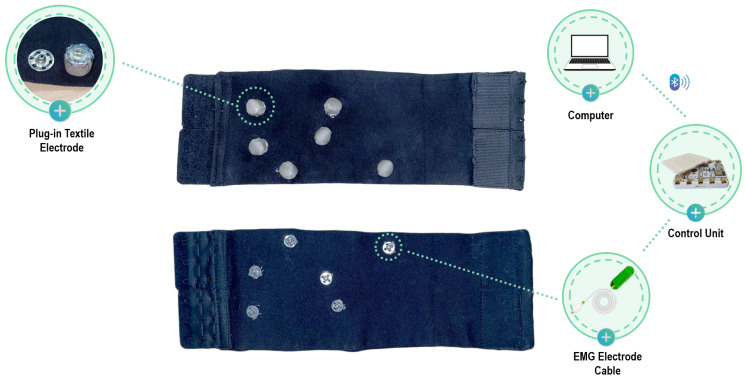
Smart textile sleeve: the upper part shows the internal view of the textile electrodes, while the lower part shows the external view connected to the acquisition system.

EMG data were collected using the assembled EMG Sensor BITalino (r)evolution BLE system (PLUX Wireless Biosignals S.A., Lisbon, Portugal) designed for real-time physiological data recording together with the OpenSignals (r)evolution software (public build 2022-05-16). The system was used both in connection to the novel wearable sleeve with e-textile electrodes and with standard Ag/AgCl electrodes for surface EMG in order to fix the acquisition protocol and focus on the differences between standard and textile electrodes. Figure 4 schematically illustrates the connection setup employed during the acquisition with the textile sleeve. The plug-in textile electrodes are attached to the internal side of the unit, while the conductive clips on the outer side are connected to the BITalino unit through a standard electrode cable. EMG signals are wirelessly transmitted to a personal computer, where they are stored and processed. Two muscles of particular interest were studied, namely, the anterior tibialis and lateral gastrocnemius. These muscles play a crucial role in human locomotion, being the major muscles responsible for foot dorsiflexion [41,42,43]. The muscles on the right side were monitored, as the participants were all right-handed. Electrodes were placed on these muscles following SENIAM recommendations [39]. EMG signals were processed in MATLAB R2023a (Mathworks Inc., Natick, MA, USA) to extract quantitative metrics estimating muscle fatigue exerted during the tasks. The parameters describing the EMG signals considered in this analysis are as follows: area of the signal, power of the signal, mean value, and Root Mean Square (RMS). These quantitative metrics were extracted from the rectified signal during the 5 s contraction windows and then averaged over a trial.

### 2.4. Statistical Analysis

In this study, a statistical analysis was conducted to compare the performances of the e-textile electrodes with the reference pre-gelled Ag/AgCl electrodes for sEMG signal acquisition. To assess whether a statistically significant difference existed between the signals acquired with the two methods, either the paired *t*-test or its non-parametric form, the Wilcoxon Mann–Whitney test, was applied according to the results of the Shapiro–Wilk test for normality of the data.

In addition, the Bland–Altman method was used to assess the agreement between the two measurement techniques. This is the most popular method for measuring agreement between two measurement systems [44]. It plots the differences between the two sets of measurements against their averages, allowing for identification of the bias (the mean difference) and the Limits of Agreement (LoA), calculated as the bias ±1.96 times the standard deviation of the differences [45,46]. If the differences between methods do not have a normal and/or symmetric distribution, then the LoAs are considered to be between the 2.5% and 97.5% percentiles. The Bland–Altman plot additionally helps to visualize any proportional or constant systematic error and to identify patterns or anomalies in the data. Significant statistical errors are said to be present if the LoA of bias does not contain any zero values. Bland and Altman suggested that the agreement between the methods being tested should be accepted if this interval contains a zero value [45]. These statistical tools provided a comprehensive framework for comparing the performance of the two types of electrodes used in this study, facilitating an understanding of their relative effectiveness in EMG signal acquisition. Statistical analyses were performed using R, version 4.0.3 (R Foundation, Vienna, Austria).

## 3. Results

Analysis of the agreement between the two measurement methods was addressed by performing the statistical tests described above. Table 1 shows all of the metrics describing the data. Descriptive statistics, i.e., the mean values and standard deviation, are provided for each parameter, each muscle, and each measurement system. The results of the statistical tests, i.e., the normality test and *t*-test for paired data, are indicated in terms of *p*-value. The level of significance was set to 0.05, with *p*-values higher than the threshold considered to be not significant (ns in the table). Paired *t*-tests were run in parametric or non-parametric form after obtaining the results of the normality test. The hypothesis of no difference between the systems was tested, with *p*-values lower than the statistical threshold suggesting the rejection of agreement between the systems and *p*-values higher than the level of significance meaning that the differences are not statistically significant and are the result of random measurement errors.

**Figure 5:**
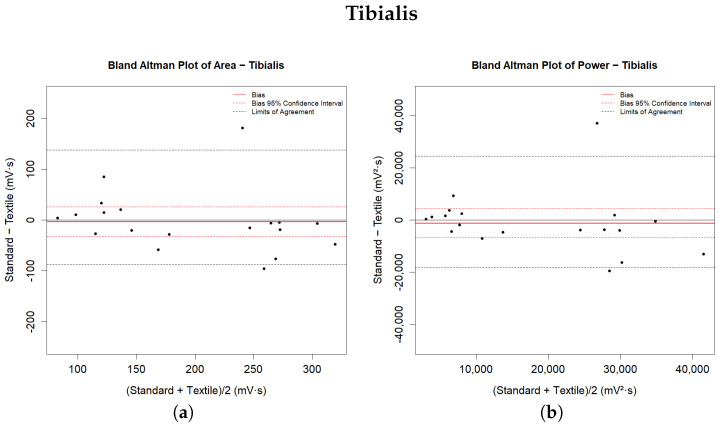

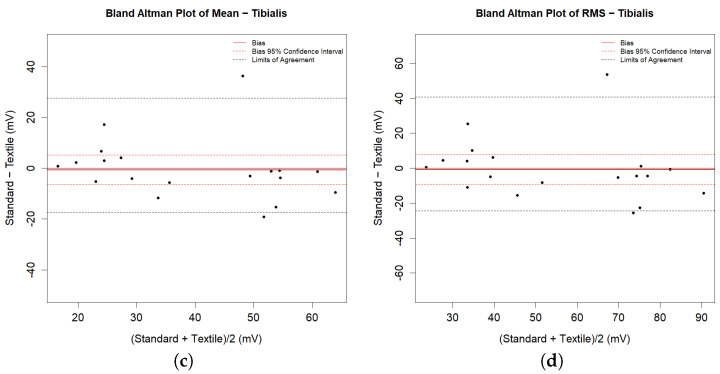
Bland-Altman Plots for parameters related to the Tibialis muscle: (**a**) Area; (**b**) Power; (**c**) Mean and (**d**) RMS parameters.

**Figure 6:**
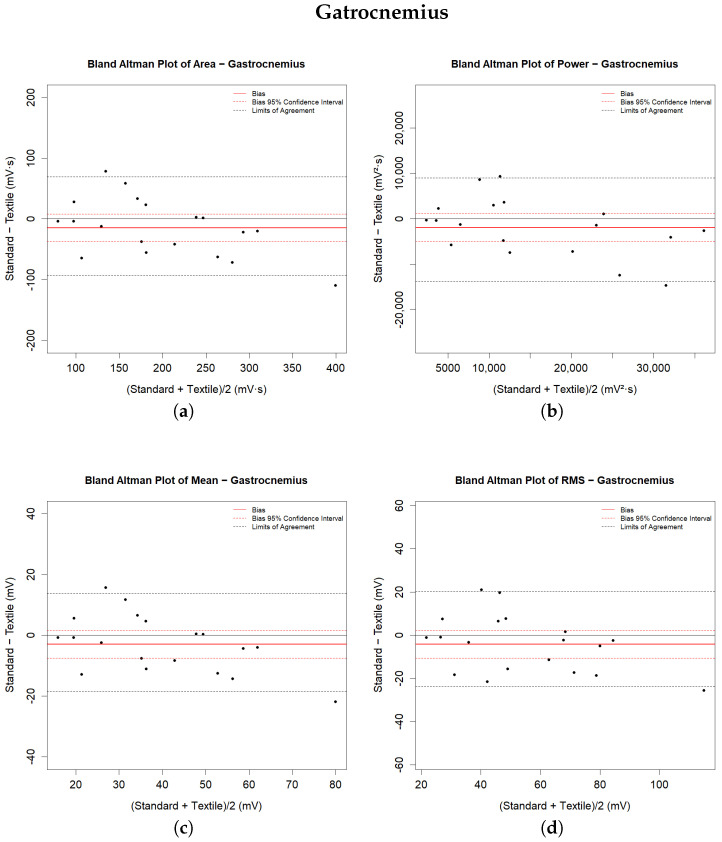
Bland–Altman plots for parameters related to the gastrocnemius muscle: (**a**) area; (**b**) power; (**c**) mean; (**d**) RMS parameters.

The same table shows the descriptive numerical values derived from the Bland–Altman analysis. The bias represents the average of the differences between the measures calculated by the systems, and is provided with the values of the LoA. In the graphs in Figure 5 and Figure 6, the bias is shown by solid red lines; the dashed red lines represent the corresponding confidence intervals. The LoA values reported in the table are shown in the graphical representations as dashed black lines. They are estimated as the 2.5 and 97.5 percentiles of the differences, as the differences do not have a symmetrical Gaussian distribution.

The results indicate general agreement for all of the analysed muscle parameters. Statistical tests for paired data did not detect a significant difference between the measurements extracted from the sEMG signals of the lower limb muscles during walking with the two measurement methods. The Bland–Altman plot shows substantial agreement between the systems, as both the confidence interval and the LoA of the bias contain zeros for all analysed parameters. The recorded differences in the extracted metrics from the signals acquired from the tibialis anterior muscle are lower than those from the lateral gastrocnemius. The recorded biases are always less than 7% of the mean parameter value for the tibialis anterior muscle and less than 12% for the gastrocnemius lateralis muscle. However, the amplitude ranges are narrower for the latter muscle than for the former. All of the biases have negative values, indicating that the signals recorded with the textile electrodes present higher magnitude and that the extracted parameters are slightly overestimated compared to those recorded with standard electrodes.

### Comfort Assessment

In addition to validating technical performance, we conducted a wearability and comfort assessment to evaluate the system’s acceptance by end users and identify areas for design improvement.

Evaluating a device’s wearability is a multidimensional analysis, as wearable devices can affect the wearer in various ways. When considering the effects of wearing something, it is important to take comfort into account. The level of comfort can be influenced by various factors, including the size and weight of the device, its impact on movement, and any discomfort it may cause.

Knight and Baber (2005) proposed that comfort should be measured across multiple dimensions, including psychological responses such as embarrassment or anxiety in addition to physical factors. To achieve this, they developed the CRS [47].

**Figure 7:**
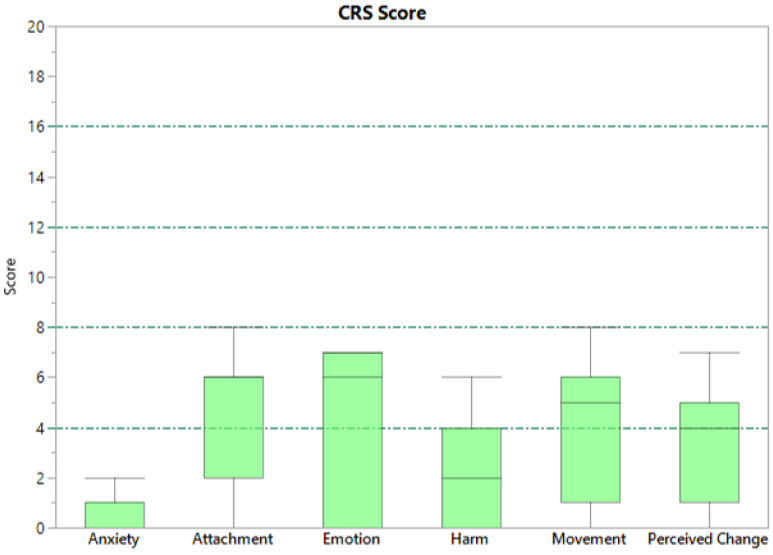
Distribution of CRS scores provided by users.

The CRS offer a convenient tool for evaluating the comfort of wearable devices. The CRS aims to provide a comprehensive assessment of the wearer’s comfort status by measuring comfort across six dimensions, as described in Table 2. To rate perceptions of comfort, the scorer indicates their level of agreement with the statements in the ‘description’ column of Table 2 on a scale from 0 (low) to 20 (high). The scores are interpreted based on the five Wearability Levels (WLs) proposed by Knight et al. (2006) [48], which are obtained by dividing the scales into equal parts. The meaning of each level is shown in Table 3. According to Knight and Baber (2005), the range used in their study was considered large enough to elicit a variety of responses that could be analysed in detail [47]. The participants in our study were invited to complete the CRS and provide their judgments. All subjects were guided by the same interviewer using standardised instructions. Figure 7 shows the scores assigned for each field by the subjects involved in our study.

The evaluation was conducted on a small sample of eleven individuals; therefore, these results should be considered preliminary. Table 2 reports the mean value and standard deviation of the scores. All of the fields were in the range of WL1, indicating that the system is wearable and changes are not required. The highest values are for attachment and movement (4.09); however this could be attributed to the wired electronic device for acquisition rather than the textile sleeve, which the users may have perceived as an embedded part of the device. Lower values were registered for the subjective perception of emotion and perceived change (3.91 and and 3.27, respectively). Subjects reported no discomfort or pain related to the use of the device (harm score 2.00). The lowest value was for anxiety (0.364), with all of the subjects indicating they felt safe using the device. The overall results confirm the high wearability and comfort of the proposed device, despite its being a prototype with low production costs. In order to better identify the device’s wearability level and ways to improve it, future analysis will aim to assess its comfort more extensively by testing it on a wider cohort of subjects.

## 4. Discussion

The current study introduces an e-textile sleeve for sEMG measurement that is designed to fit different calf sizes and to be both washable and reusable. The device integrates circular electrodes in a silver knitted fabric with a diameter of 10 mm.

For the purpose of performance analysis in measuring sEMG, this study compared the performance of the leg sleeve with pre-gelled Ag/AgCl electrodes with eleven healthy volunteers participating in MVIC testing. This methodology is in line with approaches used in recent studies exploring the effectiveness of textile electrodes in sEMG monitoring [25,26].

The results indicate good agreement between the two types of electrodes in measuring characteristic metrics of sEMG signals from the lower leg muscles. Agreement was confirmed by means of paired-data statistical tests and quantitative and qualitative analyses of Bland–Atman plots. All of the sEMG parameters demonstrate correspondence between the two measurement methods we tested. There were no statistically significant differences between the groups of measurements obtained with the two methods, and the average biases were negligible compared to the typical values of the metrics.

As discussed in the Section 1 of this work, the feasibility of sEMG measurements using dry textile electrodes has been analysed in several works that have focused mainly on the type of material used and their effect on the quality of the recorded signal. The results were generally comforting, and indicate that the use of textiles for smart clothes in biosignal detection is a solution that may have an important future.

Generally, the production process of these sensors, usually described in works focused on materials, is very complex and requires collaboration from various industries that are not accustomed to collaborating. This represents a significant barrier to large-scale production [49]. Therefore, in this study we aimed to test a commercial, readily available, and affordable fabric. The electrodes were manually created in order to demonstrate the feasibility of sEMG measurements with minimal resources and a simple production process. It seems clear from the scientific literature that the limitations are non-technical and that the acquired signal quality is satisfactory. However, a common limitation is that most studies have focused on developing new technologies and only tested the resulting devices on a single healthy subject. Only a few studies have considered larger study populations to validate the device and move towards broader diffusion of the technology. With this in mind, we expanded our analysis to eleven subjects and prioritised evaluating the wearability and comfort of the device for users. When implementing new technologies, it is important to consider the technical performance, impact on users, and applicability in real-world contexts. Our evaluation conducted through the CRS yielded positive results, despite the prototype nature and low production costs of the device. These aspects are significant, particularly considering that smart clothes for biosignal detection may replace standard methods in out-of-lab measurements where comfort and ease of use are necessary. The results of our wearability and comfort analysis indicate that the users did not experience pain from the device and suggested no significant modifications. To the best of our knowledge, no other reference studies have quantitatively investigated these aspects on wearable prototype devices for recording sEMG. Regardless, the collected answers fall within the ranges indicated by the questionnaire developers as indicating the two highest levels of wearability.

Although larger than the samples usually analysed in similar studies, the sample size in this study was still relatively small and the subjects fell within a narrow age range, which may not represent wider variations in the general population. For future research, it would be worth exploring the use of these textile sleeves in a larger and more diverse sample. As a further limitation, this study focused on four features extracted from the analysis of sEMG signals in time domain, which is a useful approach for characterising the intensity and duration of muscle activations. In future research, further interest may concern the analysis of other parameters, such as frequency content, signal-to-noise ratio, or measures of muscular fatigue. In addition, it would be interesting to evaluate the effectiveness of these electrodes in dynamic applications and under exercise conditions, which would be a suitable method for analysing muscle activation during walking or dynamic tasks. In particular, a more comfortable solution than classical methods involving adhesive electrodes and heavy instrumentation could favour remote monitoring applications, which, together with telemedicine, are growing in popularity today.

## Figures and Tables

**Table 1:** Descriptive statistics of the datasets and results of the statistical tests and analyses.

**Area (mV ms)**	**Anterior Tibialis**		**Lateral Gastrocnemius**	
	**Textile Sleeve**	**Ag/AgCl Electrodes**	**Textile Sleeve**	**Ag/AgCl Electrodes**
Mean± SD	198±90	195±76	205±100	190±76
Paired *t*-test	0.314 ns		0.186 ns	
Bias	−3.27		−14.9	
Lower LoA	−87.5		−92.8	
Upper LoA	138		69.2	
**Power (mV ms)**	**Anterior Tibialis**		**Lateral Gastrocnemius**	
	**Textile Sleeve**	**Ag/AgCl Electrodes**	**Textile Sleeve**	**Ag/AgCl Electrodes**
Mean ± SD	$1.88\cdot 10^{4}\pm 1.48\cdot 10^{4}$	$1.76\cdot 10^{4}\pm 1.26\cdot 10^{4}$	$2.01\cdot 10^{4}\pm 1.95\cdot 10^{4}$	$1.68\cdot 10^{4}\pm 1.28\cdot 10^{4}$
Paired *t*-test	0.184 ns		0.134 ns	
Bias	$-1.17\cdot 10^{3}$		$-1.92\cdot 10^{3}$	
Lower LoA	$-1.80\cdot 10^{3}$		$-1.37\cdot 10^{4}$	
Upper LoA	$2.45\cdot 10^{3}$		$9.00\cdot 10^{3}$	
**Mean (mV)**	**Anterior Tibialis**		**Lateral Gastrocnemius**	
	**Textile Sleeve**	**Ag/AgCl Electrodes**	**Textile Sleeve**	**Ag/AgCl Electrodes**
Mean ± SD	39.7±18.1	39.1±15.3	41.1±20.1	38.1±15.2
Paired *t*-test	0.314 ns		0.185 ns	
Bias	−0.649		−3.00	
Lower LoA	−17.5		−18.5	
Upper LoA	27.6		13.9	
**RMS (mV)**	**Anterior Tibialis**		**Lateral Gastrocnemius**	
	**Textile Sleeve**	**Ag/AgCl Electrodes**	**Textile Sleeve**	**Ag/AgCl Electrodes**
Mean ± SD	55.5±25.3	54.8±21.5	57±28	52.8±22.3
Paired *t*-test	0.334 ns		0.179 ns	
Bias	−0.727		−4.27	
Lower LoA	−24.4		−23.9	
Upper LoA	40.7		20.3	

**Table 2:** Description of CRS fields and results.

Title	Description	Mean	Std.
Emotion	I am worried about how I look when I wear this device. I feel tense or on edge because I am wearing the device.	3.91	3.08
Attachment	I can feel the device on my body. I can feel the device moving.	4.09	2.70
Harm	The device is causing me some harm. The device is painful to wear.	2.00	2.05
Perceived change	Wearing the device makes me feel physically different. I feel strange wearing the device.	3.27	2.41
Movement	The device affects the way I move. The device inhibits or restricts my movement.	4.09	2.74
Anxiety	I do not feel secure wearing the device.	0.364	0.674

**Table 3:** Wearability levels for interpretation of CRS scores.

Wearability Level	CRS Score	Outcome
WL1	0–4	System is wearable
WL2	5–8	System is wearable, but changes may be necessary, further investigation is needed
WL3	9–12	System is wearable, but changes are advised, uncomfortable
WL4	13–16	System is not wearable, fatiguing, very uncomfortable
WL5	17–20	System is not wearable, extremely stressful, and potentially harmful

## Data Availability

Data available on request due to privacy and legal restrictions.

## Abbreviations

The following abbreviations are used in this manuscript:

BMI	Body Mass Index
CCSM	Conductive Composite Silicone Material
CRS	Comfort Rating Scale
EMG	Electromyography
LoA	Limits of Agreement
MVIC	Maximal Voluntary Isometric Contraction
RMS	Root Mean Square
sEMG	Surface Electromyography
SENIAM	Surface Electromyography for Non-Invasive Assessment of Muscles
WL	Wearability Level
SNR	Signal-to-Noise Ratio
